# Factors Influencing Diagnostic Yield and Complication Risk in CT-Guided Transthoracic Lung Biopsy of Pulmonary Lesions: A Retrospective Analysis

**DOI:** 10.3390/diagnostics16091410

**Published:** 2026-05-06

**Authors:** Augustina Mozerytė, Juozas Jarašūnas, Kipras Mikelis, Eimantas Dumskis, Donatas Jocius

**Affiliations:** 1 Department of Radiology, Nuclear Medicine and Medical Physics, Institute of Biomedical Sciences, Faculty of Medicine, Vilnius University, 03101 Vilnius, Lithuania; juozas.jarasunas@mf.vu.lt (J.J.); kipras.mikelis@mf.vu.lt (K.M.); donatas.jocius@mf.vu.lt (D.J.); 2 Radiology and Nuclear Medicine Center, Vilnius University Hospital Santaros Klinikos, 08410 Vilnius, Lithuania; eimantas.dumskis@santa.lt

**Keywords:** CT-guided transthoracic lung biopsy, diagnostic yield, non-diagnostic results, pulmonary lesions, procedure-related complications

## Abstract

**Background/Objectives**: CT-guided transthoracic lung biopsy is a broadly performed and minimally invasive procedure for obtaining tissue samples from pulmonary lesions, offering a high diagnostic yield, and remains a cornerstone of accurate lung cancer diagnosis. The primary objective of this study was to assess the diagnostic yield of CT-guided transthoracic lung biopsies and to investigate the factors influencing this yield, with procedure-related complications being a secondary objective. **Methods**: A retrospective study was conducted on 356 CT-guided transthoracic lung biopsies performed between January 2021 and December 2023. The patient demographics, lesion characteristics (size, location) and histological findings were analyzed. The diagnostic yield was evaluated based on histological findings and, if necessary, follow-up information. **Results**: Of the 356 biopsies, 326 (91.6%) were found to be diagnostic, and 30 (8.4%) were non-diagnostic. The diagnostic yield was significantly influenced by lesion size and the number of biopsy cores, with lesions ≤2 cm associated with a higher diagnostic failure rate (*p* = 0.034). A significant interaction was also observed between lesion size and core number (*p* < 0.001). The overall complication rate was 11.5%, with pneumothorax being the most frequent event (7.0%). CIRSE grade 2 and grade 3 complications occurred in 4.8% and 3.1% of cases, respectively. Solid lesion morphology was identified as an independent predictor of complications (OR = 1.67, *p* = 0.022). **Conclusions**: In summary, CT-guided transthoracic lung biopsy demonstrates a high diagnostic yield with an acceptable safety profile and remains an important diagnostic tool in the evaluation of pulmonary lesions.

## 1. Introduction

CT-guided transthoracic pulmonary lesion biopsy (CT-TTB) is a minimally invasive procedure widely used to assess lung tissue abnormalities, especially to diagnose or categorize lung cancer. Recent advances in non-surgical lung cancer treatment require precise diagnosis and comprehensive molecular characterization of pulmonary lesions. Accurate sampling is essential for selecting targeted and personalized treatment [1,2]. CT-TTB is highly accurate, with reported accuracy rates of 90–99% [1,2]. Because it is minimally invasive, safe, and has a low complication rate, the procedure is widely accepted in clinical practice [3,4]. Notably, lesion size is one of the determinants of CT-TTB’s diagnostic performance; smaller pulmonary lesions (≤2 cm) have a lower diagnostic yield than larger ones [1,5].

CT-TTB has a higher diagnostic yield for peripheral lung nodules compared with the bronchoscopic approach. A meta-analysis of randomized controlled trials showed that CT-TTB achieved a significantly higher diagnostic yield than radial EBUS-guided transbronchial biopsy (rEBUS-TBB) (83.45% vs. 68.82%) overall, and particularly for lesions between 1 and 2 cm (83% vs. 50%) [6]. Furthermore, in patients with pulmonary consolidation, CT-TTB demonstrated a higher diagnostic yield (50.32% vs. 25.16%) and better diagnostic accuracy for malignancy than transbronchial lung biopsy (TBLB) (91.61% vs. 87.74%) [7].

Current international guidelines state that indications for lung biopsy are based on radiological features and malignancy probability, rather than solely on nodule size. The 2017 Fleischner Society guidelines recommend further evaluation, including tissue sampling, for solid nodules > 8 mm and persistent subsolid nodules with a solid component ≥6 mm, which are considered highly suspicious [8]. The British Thoracic Society and the American College of Chest Physicians similarly support a structured diagnostic approach using malignancy risk stratification to guide decisions regarding invasive procedures, including biopsy, in nodules with moderate to high probability of malignancy [9,10].

These guideline-based principles are reflected in our study cohort, as patients were selected for CT-TTB based on clinical and radiological suspicion of malignancy. Therefore, the analyzed population represents individuals in whom tissue sampling was considered clinically indicated within established diagnostic pathways. While current guidelines define when tissue sampling is indicated, they do not prescribe a single preferred biopsy modality. Instead, the choice of diagnostic approach is guided by lesion characteristics, technical feasibility, and procedural risk. Within this framework, CT-TTB represents one of the principal methods for obtaining tissue diagnosis in clinical practice.

Although minimally invasive, a transthoracic lung biopsy—like any lung biopsy—carries a risk of complications. Most occur during or shortly after the procedure [11,12]. Pneumothorax is the most common complication of CT-TTB, occurring in approximately 15–25% of cases. Only a minority of patients (4–7%) develop clinically significant pneumothorax that requires chest tube insertion or prolonged hospitalization [13,14]. Pulmonary hemorrhage is the second most common complication of CT-TTB, with reported incidence rates ranging from 4% to 27%. Hemoptysis occurs in approximately 4% of patients. Clinically significant bleeding requiring further management is rare [15,16]. In contrast, systemic air embolism remains an exceptionally rare but potentially life-threatening event, accounting for only a small proportion of procedure-related adverse outcomes [12,15,16]. To reduce the risk of procedure-related complications, standard mitigation strategies such as careful patient selection, optimization of needle trajectory, and post-procedural monitoring are routinely applied.

While CT-TTB is a well-established diagnostic technique, the combined evaluation of diagnostic yield, biopsy core number, and complication profile remains insufficiently explored in a single clinical cohort. Therefore, the aim of this study was to provide an integrated analysis of these factors and to assess their practical implications for optimizing biopsy strategy in routine clinical practice.

To our knowledge, comprehensive analyses of CT-TTB outcomes in the Baltic region are limited, and studies that integrate diagnostic yield, biopsy technique parameters, and complication profiles within a single cohort remain scarce.

## 2. Materials and Methods

### 2.1. Data Analysis

Retrospective data analysis was conducted using the institution’s database, including all consecutive patients who underwent CT-TTB in the Interventional Radiology Department during a three-year period (from 1 January 2021 to 31 December 2023). All CT-TTB procedures performed for pulmonary lesions during the study period were included in the analysis. The analysis was conducted at the level of individual biopsy procedures, with each procedure treated as a separate observation. Repeated biopsy procedures were retained, as they reflected clinically relevant follow-up interventions in cases with initially non-diagnostic or inconclusive histological results. Procedures were evaluated based on available histological outcomes, which were used to determine diagnostic yield.

Biomedical ethics approval (No. 2022/6-1441-916) was obtained for this study from the Regional Biomedical Ethics Committee. We would like to clarify that this study was designed as a single-center retrospective observational study based on routinely collected clinical data. At the time of ethics submission and approval (14 June 2022), the study protocol defined the inclusion criteria and the data source (institutional database), with the intention to include all eligible patients within the specified study period. Although the study includes data up to 31 December 2023, all data were derived from the same clinical database and were collected as part of standard clinical care, not specifically for research purposes. The ethics approval permitted access to and analysis of these data within the defined protocol, including cases recorded after the approval date, provided they met the predefined inclusion criteria. Importantly, no data were accessed or analyzed prior to obtaining ethics approval.

The collected dataset included patient demographic characteristics, such as age and sex; imaging-based lesion-related variables (lesion size, location [right or left lung; central or peripheral], and radiological characteristics); the number of biopsy cores; histological outcomes (further classified as diagnostic or non-diagnostic); and procedure-related complications. Variables included in the statistical analyses were selected based on their clinical relevance and literature analysis. Diagnostic performance was the primary endpoint, with procedural safety of CT-TTB as a secondary outcome.

For analytical purposes, age was categorized into predefined groups: ≤65 years and >65 years. Moreover, the number of biopsy cores obtained per procedure was categorized into two groups: 1–2 cores and 3–5 cores. In addition, lesion size was measured and categorized into two groups: lesions smaller than 2 cm and lesions measuring 2 cm or greater. These categorizations were based on clinically relevant thresholds and commonly used cut-offs in the literature to facilitate interpretation and comparability of results. Based on chest CT findings before biopsy, the pulmonary lesion location was determined by lung segment and was categorized as central or peripheral. Peripheral lesions are defined as those located distal to the segmental bronchi, beyond the origin of the first subsegmental bronchial branches. Lesions located at or proximal to the segmental bronchi, including those involving the hilar or peribronchovascular regions, were classified as central. Additionally, lung nodule type was classified as solid, subsolid, or ground-glass according to the 2017 Fleischner Society guidelines [8].

Based on histopathological findings, biopsy results were classified as either diagnostic or non-diagnostic. Biopsies were considered diagnostic if they confirmed malignant disease or clearly defined benign pathology without diagnostic uncertainty. Non-diagnostic biopsies were defined as those yielding insufficient or non-representative tissue, including biopsy cores containing only normal lung parenchyma or non-specific findings.

Histological findings of uncertain significance or non-specific pathology were reassessed. Final classification was based on repeat biopsy, follow-up imaging, or surgical findings. The absence of repeat biopsy or additional diagnostic procedures during 9 months of follow-up was interpreted as confirmation of the primary biopsy result as diagnostic. When follow-up evaluation (repeat biopsy, additional diagnostic investigation, or surgical intervention) subsequently confirmed malignancy, primary biopsy results were reclassified as non-diagnostic. A 9-month follow-up period was selected as a clinically reasonable interval to capture delayed diagnoses in cases with initially non-diagnostic results. Given the variability of follow-up durations reported in the literature, this timeframe was considered sufficient to identify clinically relevant diagnostic reclassification while maintaining methodological consistency within a retrospective study design.

Complications and their rates were evaluated as secondary outcomes and assessed for clinical significance. Complications were predefined and prospectively recorded. These included pneumothorax, hemoptysis, pulmonary hemorrhage, and systemic air embolism. Hemoptysis was defined as post-procedural expectoration of blood, while pulmonary hemorrhage was defined as parenchymal bleeding confirmed radiologically. All complications were categorized according to the 2017 CIRSE classification to provide a standardized evaluation of procedural safety [17].

### 2.2. Biopsy Procedure

**Figure 1:**
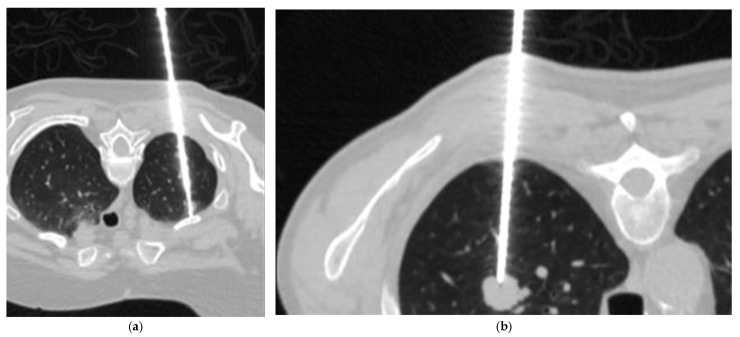
CT-guided transthoracic lung biopsy. (**a**) CT-TTB was performed with the patient in the prone position. The coaxial needle is advanced toward the target lesion, and an 18-gauge semi-automated biopsy needle is deployed through the coaxial system, as visualized on a low-dose control CT scan. (**b**) CT-TTB showing the coaxial needle positioned adjacent to the target lesion prior to tissue sampling. Following stylet removal, a biopsy was performed using an 18-gauge automated core biopsy needle.

CT-TTB biopsy was performed under low-dose computed tomography guidance. A non-contrast CT scan was used to localize the target lesion and plan the biopsy trajectory (Figure 1). After sterile preparation and administration of local anesthesia, a 17-gauge coaxial needle was advanced adjacent to the lesion, and biopsy cores were obtained using an 18-gauge needle with an automated biopsy gun or semi-automated core biopsy needle. A post-procedural low-dose CT scan was performed to assess immediate complications. Biopsy specimens were fixed in formalin and submitted for histopathological analysis. Fine-needle aspiration biopsy was not performed at this institution. All procedures were performed using a standardized core needle technique with a coaxial system.

Procedural parameters, including the number of biopsy cores, were not standardized and were determined by the performing interventional radiologist based on intra-procedural assessment.

### 2.3. Statistical Analysis

The data were analyzed using IBM SPSS Statistics for Windows, Version 20.0 (IBM Corp., Armonk, NY, USA). Frequencies and means were calculated for demographic variables. Descriptive statistics were used to describe the distribution of demographic variables, including age and sex. The accuracy, sensitivity, specificity, and diagnostic yield were calculated with 95% confidence intervals. For procedure-related complications, confidence intervals were calculated only for main complication categories (pneumothorax and hemoptysis). Other complications were reported as absolute frequencies and percentages without confidence intervals. The relationship between the number of biopsy cores and complication risk was not specifically analyzed as a primary outcome in this study. However, complication rates were evaluated in relation to lesion characteristics and other procedural factors.

Based on the obtained histological results, two groups were distinguished: the diagnostic success group and the diagnostic failure group. The diagnostic success group included true-positive and true-negative results. The diagnostic failure group consisted of false-positive, false-negative, and non-diagnostic results. False-negative results were defined as initially non-diagnostic biopsy cases that, after follow-up evaluation (repeat biopsy, surgical pathology, or radiological progression), were confirmed to be malignant. To identify independent factors associated with diagnostic outcome, multivariate logistic regression analysis was performed, including variables such as lesion size, number of biopsy cores, lesion type, and location. For regression analysis, non-diagnostic biopsy results were included in the diagnostic failure group, together with false-negative and false-positive outcomes. Associations between categorical variables were evaluated using the Chi-square or Fisher’s exact test, as appropriate. A *p*-value < 0.05 was considered statistically significant.

Missing data were not observed for the analyzed variables. All lesion characteristics were available from pre-procedural chest CT, and all procedures had corresponding histological outcomes. No formal sample size calculation was performed due to the retrospective design of the study, and all eligible procedures within the study period were included.

## 3. Results

### 3.1. Study Population Characteristics

Between 2021 and 2023, 356 patients underwent CT-TTB. The mean patient age was 65.8 ± 10.9 years (range 20–92). Women accounted for 57.6% of the cohort (*n* = 205), and men for 42.4% (*n* = 151).

Average lesion size was 18.0 mm (interquartile range [IQR] 12.0–28.0 mm). Lesions measuring ≤2 cm were observed in 208 cases (58.4%), whereas lesions larger than 2 cm were present in 148 cases (41.6%). A slightly higher proportion of lesions were located in the right lung (51.7%; *n* = 184) than in the left lung (48.3%; *n* = 172). Approximately 85% of the target lesions (*n* = 302) were peripheral, and central lesions composed only about 15% of the cases (*n* = 54). Regarding lesion characteristics, solid lesions were identified in 219 patients (61.5%), whereas subsolid, or ground-glass opacity (GGO) lesions were observed in 137 patients (38.5%).

The average number of biopsy cores obtained per procedure was 2 (range 1–5).

Baseline patient and lesion characteristics are summarized in Table 1.

### 3.2. Biopsy Results

In the study cohort, 326 of 356 biopsies (91.6%) were diagnostic. Non-diagnostic biopsies accounted for 8.4% (*n* = 30).

**Figure 2:**
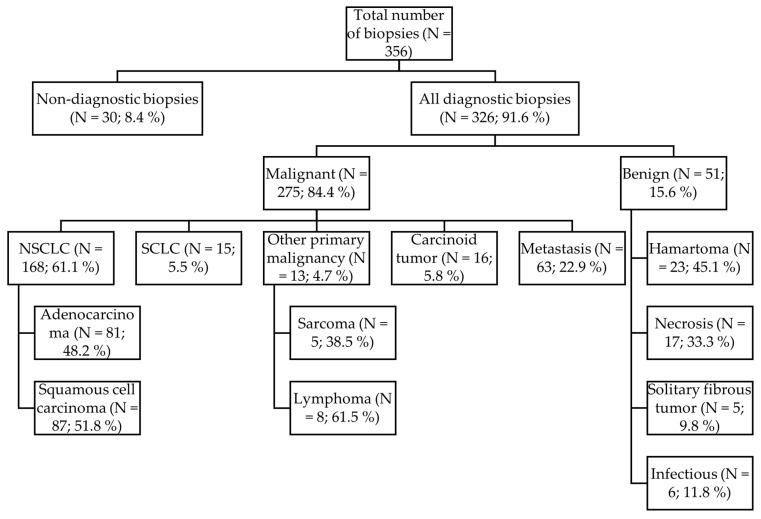
Histological results of CT-TTB.

Based on histological assessment, malignant lesions, both primary lung and metastatic tumors, comprised 84.4% (*n* = 275) of the cases, while benign conditions accounted for 15.6% (*n* = 51). The distribution of all biopsy results is detailed in Figure 2.

In this study, CT-TTB demonstrated a diagnostic yield of 91.6%. The sensitivity of the procedure was 97.9% (95% CI 95.7–99.0). Specificity was 85.2% (95% CI 67.5–94.1). The positive predictive value was 98.8% (95% CI 96.9–99.5), while the negative predictive value was 76.7% (95% CI 59.1–88.2).

### 3.3. Follow-Up Outcomes After CT-TTB

After initial CT-TTB, a definitive clinical diagnosis was not established in 77 patients. Histological analysis in 15 of these patients revealed normal lung tissue, leading to their initial classification as non-diagnostic.

The remaining 62 patients underwent a 9-month follow-up. Control chest CT was performed in 41 patients; 39 demonstrated lesion stability or regression, while 2 exhibited lesion growth. PET/CT was conducted in 3 patients, all of whom showed metabolically active lesions. Based on radiological progression and clinical information, these 5 cases were considered to represent malignancy progression and were therefore classified as false-negative initial CT-TTB results.

18 patients underwent additional diagnostic procedures. Repeat CT-TTB was performed in 6 patients, confirming malignancy in 5 cases, all of which had also been classified as false-negative initial biopsies. In the remaining case, repeat CT-TTB findings were consistent with the initial biopsy result.

Surgical biopsy was performed in 9 patients, confirming malignancy in 5 cases and demonstrating concordant benign findings in 4 cases. The malignant cases were classified as false-negative initial CT-TTB results.

Transbronchial biopsy was performed in 3 patients, with all results consistent with the initial biopsy findings.

Upon completion of all additional diagnostic procedures and radiological follow-up, 30 cases were classified as non-diagnostic CT-TTB procedures.

**Figure 3:**
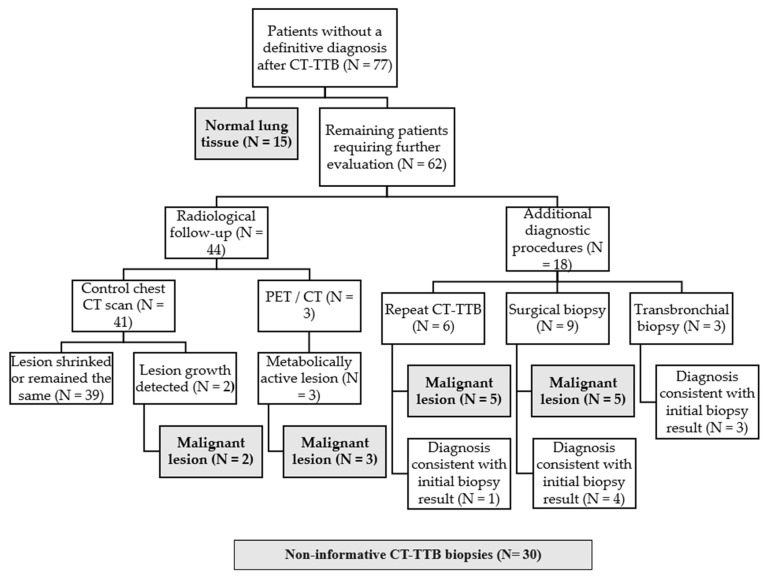
Follow-up outcomes after CT-TTB. Follow-up flowchart illustrating the clinical pathways and outcomes of patients without a definitive diagnosis after CT-TTB. Patients with initially non-diagnostic results underwent radiological follow-up or additional diagnostic procedures, including repeat biopsy or surgical intervention, to establish the final diagnosis.

A summary of follow-up outcomes after CT-TTB is presented in Figure 3.

### 3.4. Factors Associated with Diagnostic Outcomes

Lesion size smaller than 2 cm was associated with diagnostic failure (*p* = 0.034). Among lesions ≤ 2 cm, diagnostic failure accounted for 6.5% (*n* = 23) of all biopsies, while diagnostic success in this group comprised 52.0% (*n* = 185). In lesions larger than 2 cm, diagnostic failure represented 2.0% (*n* = 7) and diagnostic success 39.6% (*n* = 141) of the total biopsy cohort.

**Figure 4:**
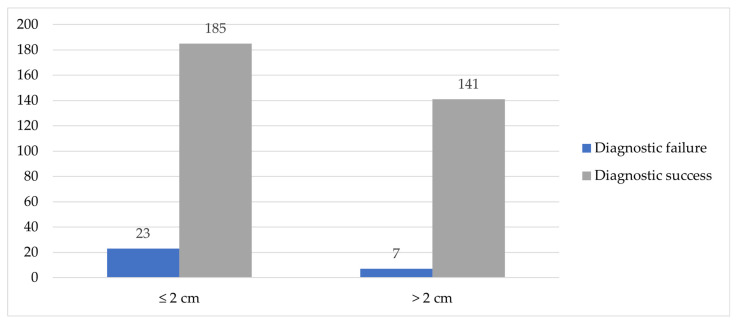
Distribution of diagnostic outcomes according to lesions size.

The distribution of diagnostic success and failure by lesion size is shown in Figure 4.

Other factors, including the radiologic characteristics of the target lesion (solid vs. subsolid/GGO; *p* = 0.441), lesion location (central vs. peripheral; *p* = 0.612), and patient age (≤65 vs. >65 years; *p* = 0.883), were not significantly associated with diagnostic outcome and were therefore considered not associated with procedural failure.

However, a statistically significant association was observed between the number of biopsy cores and diagnostic yield (*p* = 0.002). The analysis of diagnostic yield by biopsy core number is presented in Table 2. When 1–2 biopsy cores were obtained, diagnostic failure occurred in 18 of 268 procedures (6.7%), and diagnostic success occurred in 250 cases (93.3%). In contrast, procedures with 3–5 biopsy cores showed a higher failure rate (12 of 88 cases, 13.6%) and a lower success rate (76 cases, 86.4%).

**Figure 5:**
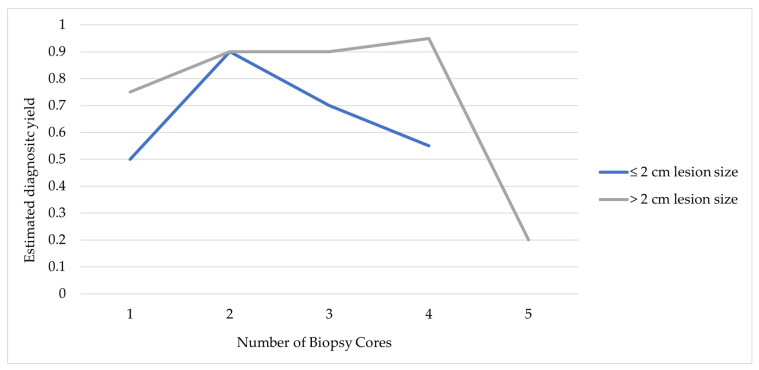
Distribution of diagnostic outcomes by lesion size and number of biopsy cores.

A statistically significant effect of lesion size and biopsy number on diagnostic yield was observed (*p* < 0.001), indicating that the effect of biopsy number on diagnostic performance varied by lesion size. As shown in Figure 5, for small lesions (≤2 cm), diagnostic yield increased substantially from one to two biopsy cores but declined with additional sampling. In contrast, larger lesions (>2 cm) maintained high diagnostic accuracy across varying biopsy numbers, with a plateau-like pattern observed after two to three biopsy cores.

To further evaluate the independence of these factors, a multivariate logistic regression analysis was performed. The results of the multivariate analysis are summarized in Table 3. Lesion size ≤ 2 cm remained an independent predictor of lower diagnostic success (*p* = 0.032, OR = 0.33, 95% CI: 0.12–0.91). Additionally, obtaining 3–5 biopsy cores remained independently associated with significantly lower odds of diagnostic success (*p* = 0.049, OR = 0.45, 95% CI: 0.21–0.99). Lesion location and morphology did not reach statistical significance in the multivariate model.

A one-way analysis of variance was performed to compare the diagnostic yield between malignant and non-malignant histological groups after excluding non-diagnostic biopsies (*n* = 326). The analysis revealed a statistically significant difference (*p* < 0.001), with malignant lesions demonstrating higher diagnostic yield. These findings indicate that, even among informative biopsies, malignant histology is considerably more likely to result in a conclusive diagnosis.

### 3.5. Complications

The overall complication rate after CT-TTB was 11.5% (*n* = 41; 95% CI: 8.0–15.0).

There were a total of 25 pneumothoraxes (7.0%; 95% CI: 4.0–10.0). According to the CIRSE classification, 2 of these cases were categorized as grade 1 complications. 15 pneumothoraxes were classified as grade 2 complications and required additional observation for less than 48 h. 8 pneumothoraxes were classified as grade 3 complications due to the need for pleural drainage and/or prolonged hospitalization exceeding 48 h.

A total of 11 cases of hemoptysis were recorded (3.1%; 95% CI: 1.7–5.6). According to the CIRSE classification, 10 cases were categorized as grade 1 complications and were successfully managed during the procedure. One case required up to 48 h of additional hospitalization and was classified as a grade 2 complication.

One case of minor hemorrhage was classified as a grade 1 complication. Another case of pulmonary hemorrhage required supplemental oxygen therapy and was categorized as a grade 2 complication. Two cases were categorized as grade 3 complications and required admission to the intensive care unit due to massive bleeding.

One patient experienced a rare and serious complication—systemic air embolism involving the coronary arteries. In this case, the complication occurred after biopsy of a 13 mm peripheral lesion in the right upper lobe. The patient developed acute weakness, pallor, and diaphoresis. Control CT imaging demonstrated air within the coronary arteries. The patient was managed conservatively with Trendelenburg positioning, supplemental oxygen, and close monitoring. No electrocardiographic abnormalities were observed, and the patient’s clinical condition improved without the need for further intervention.

Complications and their grades according to CIRSE are summarized in Table 4.

Multivariate logistic regression analysis identified lesion type (solid vs. subsolid/ground-glass) as the only variable significantly associated with complication rate.

Solid lesions demonstrated a 1.7-fold increased likelihood of any complication (*p* = 0.022, OR = 1.67) and twice as likely to result in pneumothorax (*p* = 0.002, OR = 1.97).

Other variables, such as patient age (*p* = 0.097), lesion size (*p* = 0.491), and lesion location (*p* = 0.642), were not statistically significant predictors of procedure-related complications.

## 4. Discussion

Given its high sensitivity and specificity, CT-TTB has become a widely used diagnostic approach for evaluating pulmonary lesions, particularly those located peripherally [5]. Compared with bronchoscopic techniques, CT-TTB generally provides a higher diagnostic yield for these nodules, whereas bronchoscopic approaches are associated with lower complication rates. Therefore, the choice of diagnostic modality should be individualized based on lesion characteristics and clinical context.

From a clinical decision-making perspective, CT-TTB and bronchoscopic approaches should be considered complementary rather than competing techniques. CT-TTB is particularly advantageous for peripheral pulmonary lesions, especially those smaller than 2 cm, for which bronchoscopic approaches demonstrate a significantly lower diagnostic yield [6]. In contrast, bronchoscopic techniques are generally preferred for centrally located lesions or when a lower complication risk is a priority [6,7]. Therefore, in clinical practice, CT-TTB may be considered the first-line diagnostic approach for peripheral lesions with high suspicion of malignancy, while bronchoscopic biopsy remains an important alternative for central lesions, in cases of higher procedural risk, or when endobronchial access is feasible. This risk-adapted and lesion-oriented approach is consistent with current guideline-based diagnostic pathways [8,9,10].

In this retrospective study, the diagnostic yield for pulmonary lesions was 91.6%, aligning with previous studies reporting a diagnostic yield of 85–95% [18]. The sensitivity of CT-TTB was 97.9%, which is consistent with the 92% to 97% range reported in the literature and confirms its strong ability to detect malignancy. The specificity was 85.2%, which is comparatively lower. This likely reflects a well-known limitation of CT-TTB in excluding malignancy in inconclusive cases rather than suboptimal method performance. The results showed a high positive predictive value (98.8%), confirming the method’s ability to detect malignancy and supporting PPVs of 99.2% to 99.4% in multicenter studies [19]. The relatively low negative predictive value (76.7%) reflects an inherent limitation of CT-TTB in reliably excluding malignancy. This is particularly relevant in cases with non-diagnostic histological findings. This highlights that a negative biopsy result does not definitively exclude malignancy and should be interpreted in the context of clinical and radiological findings. Therefore, continued follow-up and, when appropriate, repeat biopsy or alternative diagnostic approaches are essential to avoid delayed diagnosis.

Non-diagnostic cases accounted for 8.4% of the cohort. Lesion size was an important determinant of diagnostic yield. Biopsies from lesions measuring ≤2 cm were significantly more likely to result in diagnostic failure (*p* = 0.034). In addition, multivariate logistic regression confirmed that lesion size ≤ 2 cm remained independently associated with lower odds of diagnostic success (*p* = 0.032, OR = 0.33). This observation is consistent with previous studies showing that smaller nodules are associated with higher biopsy failure rates, likely due to technical challenges and reduced tissue adequacy. Several studies have demonstrated that smaller pulmonary nodules complicate accurate needle placement and are more likely to result in insufficient tissue samples [1,20,21].

In our cohort, lesion morphology (solid vs. subsolid/GGO) was not significantly associated with diagnostic yield (*p* = 0.441). This finding may be explained by clinical selection bias, as CT-TTB is typically performed in lesions that are already considered suspicious based on imaging characteristics, resulting in a relatively homogeneous higher-risk study population and limiting detectable differences between morphological subtypes.

From a clinical perspective, biopsy performance should be interpreted within a broader risk-adapted diagnostic workflow. Subsolid and GGO nodules are frequently managed with imaging-based surveillance, whereas tissue sampling is generally reserved for nodules demonstrating higher-risk features, such as interval growth or the development of a solid component [22].

In addition, lesion morphology may influence biopsy performance. Subsolid and GGO lesions may present greater sampling challenges due to their lower cellular density and less well-defined structure, which may contribute to non-diagnostic results.

Both lesion size and the number of biopsy cores were associated with diagnostic yield (*p* < 0.001). Diagnostic success was achieved in 93.3% of cases when 1–2 cores were obtained, compared with 86.4% when 3–5 cores were taken. However, the observed association between a higher number of cores and lower diagnostic success should be interpreted with caution. This finding is unlikely to reflect a causal relationship but rather increased procedural complexity, as additional biopsy cores are typically obtained in technically challenging cases or when initial cores are considered insufficient. As shown in Figure 5, diagnostic yield was highest when two biopsy cores were obtained, particularly in smaller lesions. This observation may be influenced by the same underlying factors, as the number of cores obtained was not standardized.

Although increasing the number of cores did not appear to improve diagnostic yield in this cohort, obtaining at least two cores may still be important to ensure adequate tissue sampling for histological and molecular analysis [18,23]. Previous studies have demonstrated high tissue adequacy rates (>95%) for CT-TTB, particularly when at least two cores are obtained [18,23]. These findings suggest that the number of biopsy cores should be tailored to procedural conditions rather than applied as a fixed strategy.

Among diagnostic biopsies, malignant lesions predominated, accounting for 275 cases (84.4%), whereas benign pathology was observed in 51 cases (15.6%), with hamartoma being the most common. Non-small cell lung cancer accounted for 168 cases (61.1%), of which adenocarcinoma was the commonest (*n* = 81, 48.2%), whereas squamous cell carcinoma was observed in 87 cases (51.8%). The presence of malignant pulmonary lesions was a factor influencing diagnostic yield (*p* < 0.001). These findings are consistent with previous studies showing higher diagnostic yield in malignant lesions. Single-center retrospective studies have shown a high diagnostic yield of malignant pulmonary lesions, whereas benign lesions have shown a higher rate of non-diagnostic or false-negative results [21]. In a meta-analysis by Wu et al., which included 30 studies with over 5000 patients, CT-TTB demonstrated a very high sensitivity of 94% and specificity of 89% for malignancy, whereas the diagnostic yield was lower for benign lesions, which required further investigation [13].

The overall complication rate was 11.5%. Most of the complications, according to the CIRSE classification, were grade 1, which were treated within the same session. There were fewer grade 2 and grade 3 complications. Pneumothorax requiring additional observation or intervention (grade 2–3) occurred in 6.5% of patients, while hemoptysis requiring additional management (grade 2) was noted in 0.3%. These results are consistent with previously reported rates of pneumothorax requiring clinical management [13,14] and extend evidence that higher-grade bleeding is uncommon after CT-TTB [15,16]. A rare but serious complication—systemic air embolism—was observed in one case. Although its incidence is low, this complication is a potentially life-threatening event. Its occurrence underscores the need for strict adherence to procedural techniques, careful needle positioning, and heightened operator awareness during CT-TTB procedures.

Pneumothorax is the most frequently reported complication of CT-TTB, with overall incidence rates ranging from approximately 15% to 40% [14,16,24,25]. Although it is consistently identified as the most common adverse event after CT-TTB, small volumes of air within the pleural cavity are often detected and are typically clinically insignificant [14]. Moreover, minimal or asymptomatic pleural air collections may not require intervention. Recent literature has emphasized the importance of distinguishing true complications from expected procedural consequences, suggesting that minimal, asymptomatic pneumothorax should not necessarily be classified as an adverse event [26]. In addition, the literature suggests that several pleural breaches are associated with an increased risk of complications [16,24,25]. In the present study, the use of a coaxial technique may have contributed to the relatively low complication rate. This approach allows multiple tissue samples to be obtained through a single pleural puncture, thereby reducing the number of pleural passes and limiting repeated lung injury. As a result, the risk of complications such as pneumothorax and bleeding may be reduced.

In our study, neither patient age, lesion size, nor location was significantly associated with complication risk. However, while lesion morphology was not associated with diagnostic yield, it emerged as a significant predictor of procedural complications in our cohort. Solid pulmonary lesions were associated with a 1.67-fold increased risk of overall complications (*p* = 0.022) and a 1.97-fold higher likelihood of pneumothorax (*p* = 0.002). These results contrast with recent large-scale data by Lee et al., which observed similar pneumothorax rates across solid, subsolid, and non-solid lesion types but reported an increased risk of pulmonary hemorrhage in subsolid nodules [27]. This evidence suggests that pulmonary lesion type does not consistently influence overall procedural risk.

This study has several limitations. First, its retrospective design based on routinely collected clinical data did not allow for prospective standardization of biopsy technique or sampling strategy. The interventional radiologist determined the number of biopsy cores, which may have introduced operator-dependent variability and potential confounding.

Moreover, specific procedural factors such as needle path length or fissure crossing, which are known to influence complication risk, were not systematically recorded and therefore could not be included in the analysis.

In addition, the categorization of continuous variables such as lesion size and age may have reduced statistical precision. Patient-reported outcomes were not assessed, limiting evaluation of the broader clinical impact of the procedure.

Second, as a single-center study, the findings may not be fully generalizable to other institutions with different patient populations and procedural protocols. Selection bias cannot be excluded, as only patients referred for CT-TTB were included.

Finally, although a 9-month follow-up period was used to classify non-diagnostic biopsies, this duration may be insufficient to detect slow-growing or indolent lesions, and delayed diagnoses beyond this interval cannot be completely excluded.

Building on these considerations, this study provides a comprehensive evaluation of CT-TTB performance by jointly analyzing diagnostic yield, histological outcomes, biopsy technique parameters, and complication risk in a real-world clinical setting, thereby offering clinically relevant insights for optimizing biopsy strategies in routine practice.

## 5. Conclusions

CT-guided transthoracic lung biopsy has a high diagnostic success rate (91.6%), acceptable sensitivity (97.9%) and specificity (85.2%). Malignant lesions had a significantly higher diagnostic success rate than benign lesions, emphasizing the technique’s value for cancer staging. Lesions smaller than 2 cm and obtaining 3–5 biopsy cores were independent predictors of lower diagnostic success rates. In this cohort, diagnostic yield was highest when two cores were obtained; however, the association between a higher number of cores and lower diagnostic success likely reflects increased procedural complexity rather than a direct causal relationship.

CT-guided transthoracic lung biopsy demonstrated an acceptable safety profile, with mostly CIRSE grade 1 complications. Higher-grade complications were less common, and severe complications were rare. Lesion morphology was the only independent predictor. However, given inconsistent findings reported in the literature, the influence of lesion type on overall procedural safety appears variable.

Lesions ≥ 2 cm were associated with higher diagnostic yield, particularly in malignant cases. Tailored biopsy planning based on lesion characteristics may improve diagnostic accuracy, reinforcing the importance of guideline-based practice.

## Figures and Tables

**Table 1:** Baseline characteristics of the study cohort.

Variable	Total (*n* = 356)
Age, years	65.8 ± 10.9 (range 20–92)
Age ≤ 65 years	164 (46.1%)
Age > 65 years	192 (53.9%)
Sex	
Female	205 (57.6%)
Male	151 (42.4%)
Lesion size, mm	18.0 [12.0–28.0]
Lesion size ≤ 2 cm	208 (58.4%)
Lesion size > 2 cm	148 (41.6%)
Lesion location	
Peripheral	302 (84.8%)
Central	54 (15.2%)
Lesion type	
Solid	219 (61.5%)
Subsolid/GGO	137 (38.5%)
Number of biopsy cores	2.0 [1.0–5.0]

**Table 2:** Relationship between the number of biopsy cores and diagnostic success.

Number of Biopsy Cores	Diagnostic Failure	Diagnostic Success	Total	*p* = 0.002
1–2 biopsy cores	18 (6.7%)	250 (93.3%)	268	
3–5 biopsy cores	12 (13.6%)	76 (86.4%)	88	
Total	30	326	356	

**Table 3:** Multivariate logistic regression analysis of factors associated with diagnostic success.

Factor	B	S.E.	Wald	*p*-Value	OR (95% CI)
Lesion size (≤2 cm)	−1.109	0.518	4.582	**0.032**	0.33 (0.12–0.91)
Biopsy cores (3–5)	−0.791	0.402	3.868	**0.049**	0.45 (0.21–0.99)
Lesion type (Solid)	0.730	0.512	2.031	0.154	2.07 (0.76–5.66)
Location (Peripheral)	0.612	0.574	1.135	0.287	1.84 (0.60–5.67)

Bold values indicate statistical significance (*p* < 0.05).

**Table 4:** Complication rates classified by CIRSE.

Complication According to CIRSE Classification	Complication	Frequency (% of All Biopsies)
1—complications managed during the same procedure, without the need for additional medical therapy and with no deviation from the normal hospitalization course.	Pneumothorax	2 (0.6%)
	Hemoptysis	10 (2.8%)
	Hemorrhage	1 (0.3%)
2—Additional observation (<48 h), no further interventions required after the procedure.	Pneumothorax	15 (4.2%)
	Hemoptysis	1 (0.3%)
	Hemorrhage	1 (0.3%)
3—Additional interventions required after the procedure, with prolonged hospital stay (>48 h).	Pneumothorax	8 (2.2%)
	Hemorrhage	2 (0.6%)
	Coronary air embolism	1 (0.3%)

## Data Availability

The datasets used and/or analyzed during the current study are available from the corresponding author upon reasonable request.

## Abbreviations

The following abbreviations are used in this manuscript:

CT-TTB	CT-guided transthoracic lung biopsy
rEBUS-TBB	radial EBUS-guided transbronchial biopsy
TBLB	transbronchial lung biopsy
CT	Computed tomography
GGO	Ground-glass opacity
NSCLC	Non-small cell lung cancer
SCLC	Small cell lung cancer
